# Extraanatomical coronary artery bypass grafting in patients with severely atherosclerotic (Porcelain) aorta

**DOI:** 10.1186/1749-8090-8-86

**Published:** 2013-04-15

**Authors:** Gokce Sirin, Kamil Sarkislali, Murat Konakci, Ergun Demirsoy

**Affiliations:** 1 Department of Cardiovascular Surgery, Goztepe Medical Park Hospital, E5 Uzeri 23 Nisan Sok. No: 17 Merdivenkoy Kadıkoy, Istanbul, Turkey; 2 Department of Anesthesiology, Goztepe Medical Park Hospital, E5 Uzeri 23 Nisan Sok. No: 17 Merdivenkoy Kadıkoy, Istanbul, Turkey

**Keywords:** Aortic diseases, Atherosclerosis, Porcelain aorta, Coronary artery bypass grafting

## Abstract

**Background:**

Cannulation, cross clamping, or partial clamping of the aorta during a proximal anastomosis may cause embolic complications in patients with severely atherosclerotic (porcelain) aortas. These patients carry high morbidity and mortality risks due to intraoperative atheroembolism.

**Methods:**

Between June 2008 and May 2010, 972 open heart surgery operations were performed in our department. In this group there were 41 patients who had severe atherosclerotic plaques in the aorta (porcelain aorta), and 9 of these underwent an extraanatomical coronary artery bypass grafting (CABG). These 9 patients were retrospectively analyzed and their demographic data, patient risk factors, and preferred surgical methods were reviewed.

**Results:**

Seven patients underwent two-vessel CABG, while 2 underwent three-vessel CABG. Off-pump surgery was performed for 7 patients. CABG was performed with beating heart technique under cardiopulmonary bypass via femoral artery and right atrial cannulation without cross clamping in 2 of the patients. Postoperative course was uneventful in all patients. Mean length of stay in the intensive care unit was 2.11 ± 0.78 days. Mean hospitalization was 7.22 ± 0.97 days. Mean follow-up was 11.33 ± 3.67 months, and no cerebrovascular events were observed during this period. Postoperative evaluation of the grafts by multislice computed tomography revealed sufficient patency in all patients.

**Conclusions:**

Innominate artery is an alternative inflow source for the untouchable ascending aorta caused by severe atherosclerotic disease (porcelain aorta). In this group of patients, the risk of systemic embolisation and perioperative neurologic complications can be minimized by avoiding manipulation of the ascending aorta and using the innominate artery.

## Background

Severely atherosclerotic aorta is a potent risk factor for perioperative atheroembolism, causing increased morbidity and mortality during cardiac operations [1-3]. The porcelain or unclampable aorta presents a difficult problem to the cardiac surgeon. Clamping of an extensive or totally calcified aorta carries a high risk of cerebral embolism. The importance of systemic embolism originating from a severely atherosclerotic ascending aorta and the associated high incidence of cerebral vascular accidents after cannulation and clamping in these aortas are well documented [4].

The incidence of significant atherosclerosis of the ascending aorta in patients undergoing cardiac surgery have been reported to be between 14% and 29% in recent studies [5-7]. The incidence of porcelain or unclampable aorta, where the aorta cannot be used as the inflow portion for any conduit, ranges between 0.4% to 5.4% according to different series [8-11]. A strong correlation between atheroembolism and atherosclerosis of the ascending aorta was documented at autopsy by Blauth and coworkers who showed that most of the patients who had evidence of atheroemboli and died after cardiac surgery had extensive atherosclerosis of the ascending aorta [12].

Currently most surgeons perform cardiac surgery by the differential application of a fully occluding cross clamp to construct the distal anastomosis and a partially occluding clamp to construct the proximal anastomosis. Severe atherosclerosis of the ascending aorta or "porcelain aorta" requires technical modifications in cardiopulmonary bypass (CPB) as well as revascularization techniques. Various techniques have been described to prevent atheroembolism caused by manipulation of extremely calcified ascending aortas. These techniques include full pedicle arterial revascularization with hypothermic fibrillatory arrest in combination with cannulation of distal arterial sites [13] and use of the innominate or axillary arteries [2,3].

The present report describes our effort to prevent atheroembolic events in patients with severely calcified ascending aortas who underwent extraanatomic coronary artery bypass grafting (CABG) procedures.

## Methods

Between June 2008 and May 2010, 972 open heart surgery operations were performed in the Department of Cardiovascular Surgery of our institution. The study was approved by the Local Ethics Committee of Goztepe Medical Park Hospital. All patients were operated by the same surgical teams. In this group there were 41 patients who had severe atherosclerotic plaques in the aorta (porcelain aorta), and 9 of these underwent an extraanatomical CABG. The innominate arteries were used for the proximal anastomotic site in these patients. Registry database, medical notes and charts were studied for preoperative and postoperative data of the patients. The indication for surgical revascularization was in accordance with international guidelines. No randomization was involved and the patient cohort was reviewed retrospectively. This study included only patients in whom the innominate artery was used for the proximal anastomotic site with left internal mammarian artery (LİMA) to left anterior descending artery (LAD) bypass. Patients who underwent surgery using only pedicle arterial grafts, LIMA to LAD artery anastomosis, and T-grafts were excluded.

The ages of the patients were described as mean standart deviation, and other data were given as frequencies or percentages. Calcified ascending aorta was noticed in the preoperative period in two patients by chest X rays (both a chest X ray and computed tomography were taken in these two patients), while it was noticed after median sternotomy by palpation of the ascending aorta in the remaining 7 patients. In all cases, the degree of atherosclerosis of the ascending aorta was graded as severe according to the classification made by Mills and Everson [4]. To avoid cross-clamping of the diseased ascending aorta, 7 patients were operated on the beating heart and 2 patients were operated on the beating heart under supportive CPB without fibrillation.

### Surgical technique

Following median sternotomy the pericardium was opened, and the aorta was palpated gently. When a calcific plaque was felt during palpation a safe site for cannulation was not searched further. After the conduits were harvested, heparin was administered (300 IU/kg). The activated clotting time was monitored and maintained longer than 300 seconds during the procedure. Innominate artery, which was selected as the site of proximal anastomosis, was also palpated for atherosclerotic plaques. Innominate artery was the inflow source for all patients. The surgical procedure was conducted via beating heart procedure (off-pump surgery) in 7 patients. The heart was positioned with posterior pericardial retraction sutures and stabilized by using a mechanical suction stabilizer (Medtronic Octopus® 2). In the other two patients, surgery was performed by beating heart technique under supportive CPB. Right femoral artery and right atrial cannulation was performed, but without cross clamping and fibrillation. A moderate hypothermia (32^°^C) was established. All distal anastomoses were performed without aortic cross-clamping. Local perfusion was maintained during each anastomosis using intraluminal shunts. All revascularization procedures were performed using internal thoracic arterial grafts and reversed saphenous vein grafts. Innominate artery was the inflow source and was partially clamped if no atherosclerotic plaques were seen. Then, proximal anastomoses were made with continuous 6.0 polypropylene suture under partial clamping of the innominate artery. The conduits used for revascularization are shown in Table 1.

**Table 1:** Operative variables of the patients

**Patients**	**Surgical procedure, inflow-target vessel**	**Surgical technic**
1	CABGx2	Beating heart
	Innominate artery-LAD (SVG)- Circumflex artery (First Obtuse Marginal branch) (SVG)	
	(Y-Graft)	
2	CABGx2	Beating heart
	LIMA-LAD, Innominate artery-acute margin artery (SVG)	
3	CABGx2	Beating heart
	LIMA-LAD, Innominate artery-RCA (SVG)	
4	CABGx2	Beating heart
	LIMA-LAD, Innominate artery-RCA (SVG)	
5	CABGx2	Beating heart on CPB
	LIMA-LAD, Innominate artery-Circumflex artery (Second Obtuse Marginal branch) (SVG)	
6	CABGx3	Beating heart on CPB
	LIMA-LAD, Innominate artery-RCA (SVG)-Circumflex artery (Third Obtuse Marginal branch) (SVG) (Y-Graft)	
7	CABGx2	Beating heart
	LIMA-LAD, Innominate artery-Circumflex artery (First Obtuse Marginal branch) (SVG)	
8	CABGx2	Beating heart
	LIMA-LAD, Innominate artery-Right posterior descending artery (SVG)	
9	CABGx3	Beating heart
	LIMA-LAD, Innominate artery-acute marjin artery (SVG)- Circumflex artery (First Obtuse Marginal branch) (SVG) (Y-Graft)	

CABG: Coronary artery bypass grafting, LAD: Left anterior descending artery, SVG: Saphenous vein graft, LIMA:Left internal mammarian artery, RCA: Right coronary artery, CPB: Cardiopulmonary bypass.

## Results

Demographic data are summarized in Table 2. All patients were male except for one, and mean age was 74,44 ± 6,39. The most common risk factor was smoking, which was present in 77.8% of the patients, and was followed in order by hypertension, diabetes mellitus, chronic obstructive pulmonary disease (COPD) and peripheral arterial disease. Two patients had three vessel, and seven patients had two vessel disease. The preoperative ejection fraction was above 50% in 33.3% of the patients.

**Table 2:** Demographic variables of patients

	**n (%)**
Age*	74,44 ± 6,39
Male/Female	8/1
Co-morbidities	
Diabetes Mellitus	5 (55,6%)
Hypertension	6 (66,7%)
COPD	3 (33,3%)
Peripheral arterial disease	3 (33,3%)
Smoking	7 (77,8%)
Coronary Artery Disease	
Double vessel	7 (77,8%)
Triple vessel	2 (22,2%)
Left ventricular EF	
>50%	3 (33,3%)
30-50%	6 (66,7%)

*. Mean ± satandard deviaton, COPD: chronic obstructive pulmonary disease, EF: ejection fraction.

A LIMA-LAD anastomosis was performed in 8 patients, and bypass to the LAD was made with a saphenous vein in one patient. The distal anastomotic sites other than the LAD were the right coronary artery in 6 (body in 3, acute marginal branch in 2, right posterior descending branch in 1), and circumflex artery in 5 (first obtuse marginal branch in 3, second obtuse marginal branch in 1, third obtuse marginal branch in 1) (Table 1). Innominate artery was the proximal anastomotic site in all patients. The revascularization procedures are summarized in Table 1.

In 7 patients the CABG procedure was made with the beating heart technique. In 2 patients the surgical procedure was performed with the beating heart technique under CPB, using femoral arterial and right atrial cannulation without cross clamping. Total CPB times in these two patients were 47 minutes and 86 minutes. No ischemic complications due to femoral arterial cannulation (lower limb ischemia) were encountered.

No hemodynamic instability was seen in the early postoperative period. The whole postoperative course was free of any cerebrovascular events and any of its signs.

The mean length of stay in the intensive care unit was 2.11 ± 0.97 days. The mean length of stay in the hospital was 7.22 ± 0.97 days. The mean follow-up time was 11.33 ± 3.67 months, and no cerebrovascular events were observed during this period. Postoperative evaluation of the grafts by multislice computed tomography revealed sufficient patency in all patients.

## Discussion

Atheroembolism has remained as the main source of cerebrovascular events for years. Atherosclerotic disease of the ascending aorta carries a higher risk of atheroembolism due to manipulation of the aorta during cardiac surgery. Heavily calcified "porcelain" aorta is associated with increased morbidity and mortality during any cardiac surgery because of increased risk of atheroembolism. The number of patients who require avoidance of manipulation of the ascending aorta increases every year. Avoiding manipulation of such an aorta is crucial in preventing cerebrovascular events. A conventional CABG procedure is performed by: (1) cannulation of the aorta, (2) cross-clamping of the aorta and (3) partial clamping of the aorta for construction of the proximal anastomosis. These manipulations have been proposed as the main sources of atheroembolism [13].

The most common sites for cannulation during conventional cardiac surgery are the ascending aorta or femoral arteries [4,6,13-16]. The severely atheromatous ascending aorta precludes conventional arterial cannulation or clamping. Manipulation of diseased aorta may result in atheroembolism [13,16-18]. Digital palpation with a lowered systemic blood pressure can be used to get a softer spot in the aortic arch for cannulation. Also, epiaortic echocardiography can be used to confirm the atherosclerotic spots and it is superior to both manual palpation of the ascending aorta and transesophageal echocardiography (TEE) in the detection of atherosclerosis of the ascending aorta, especially the noncalcified type [5,6,19-23]. The frequency of cerebrovascular events after cardiac surgery due to cerebral embolism may be reduced using epiaortic echocardiography which detects the atherosclerotic aortic sites and prevents ascending aortic manipulations (e.g., cannulation for CPB, cross clamping and partial clamping of the aorta, and construction of proximal anastomoses onto the aorta) [19,24-26]. Intraoperative TEE has routinely been used for patients with valvular diseases. None of the patients included in our study underwent TEE examination. After median sternotomy and opening of the pericardium, the aorta was palpated gently in all patients. When an atheromatous ascending aorta or heavily calcified "porcelain" aorta was detected, a safe site for cannulation was not searched further.

In conditions where the ascending aorta cannot be cannulated safely due to atherosclerotic diseases, use of a peripheral cannulation (femoral or axillary artery) is suggested [16,19]. Coexistence of severe abdominal aortic atherosclerosis and atherosclerosis of iliofemoral arteries in patients with coronary artery disease can result in the inability to use the femoral arteries as cannulation site [4,5,12-15,27]. Axillary artery is an alternative site for arterial cannulation and is more desirable than the femoral artery since retrograde blood flow carries a high risk of retrograde atheroemboli [13,16,19,28]. In this study, femoral artery cannulation was preferred in two patients because the diameter of the femoral artery was better than the axillary artery. We also did not encounter any ischemic events caused by femoral artery cannulation.

Various techniques have been described to reduce the risk of atheroembolism that may cause cerebrovascular events in patients with atherosclerotic ascending aorta. These include "no-touch" technique [10], single-clamp technique [14,26], placement of proximal saphenous vein (SV) grafts onto the internal mammary artery (IMA), onto the innominate artery [4,5,9,13,15], onto the axillary artery, [9,29] or onto the carotid artery [27,30,31], complete arterial revascularization with pedicle arterial grafts (ITA and right gastroepiploic artery) under hypothermic fibrillatory arrest that avoids clamping of the ascending aorta [10,13,32], replacement of the ascending aorta [24], aortic endarterectomy [17,19,33], patch aortoplasty, and arterial cannulation of the axillary artery [13,16,28]. Furthermore, coronary-coronary bypass on a beating heart (off-pump surgery) can be performed safely in patients with porcelain aorta [34].

Holland and Hieb [35] and Weinstein and Killen [15] have both individually reported use of the innominate artery as the inflow vessel. Bonatti et al [36]. reported acceptable graft patencies in patients with severely atherosclerotic ascending aortas and redo operations using the subclavian and axillary artery as inflow vessels. Demirsoy et. al [9]. performed extraanatomic bypass operations to 8 patients with porcelain aorta, and preferred the innominate artery in 6 patients, axillary artery in 1 patient, and the subclavian artery in 1 patient as the site of proximal anastomosis. Postoperative graft patency was assessed with contrast enhanced electron beam coronary angiography, and only one saphenous vein graft to the right coronary artery was observed to be occluded. In the present report, the innominate artery was used as the inflow source to construct the proximal anastomosis in all patients. The patients were followed up for a mean period of 11.33 ± 3.67 months, and all grafts remained patent during this time.

The beating heart technique is the method of choice for treating patients with porcelain aortas undergoing CABG. This technique avoids aortic manipulation and reduces the risk of a postoperative embolic cerebrovascular event [5,37]. However, revascularization of the circumflex coronary artery system in patients with multivessel disease may be difficult on the beating heart. Although hypothermic circulatory arrest has also been suggested as an alternative, it is not without complications [16,37]. We performed coronary artery bypass grafting procedure on the beating heart in all 9 patients. Because they were hemodynamically unstable, in two patients the surgical procedures were performed with the beating heart technique under supportive CPB and without using cross clamping and fibrillation. The atherosclerotic aorta was not used as a site of proximal anastomosis. There was no need for cross-clamping of the aorta and infusion of cardioplegic solution. Since the innominate artery was the site of proximal anastomoses, no partial clamp was placed on the atherosclerotic aorta. When the time required for cooling and heating is considered in patients who undergo hypothermic circulatory arrest, this appears as a straightforward method that does not prolong procedural time. With these strategies, we were able to avoid embolization from the severely atherosclerotic ascending aorta and prevent any embolic cerebrovascular event, as would have been expected in association with manipulation of calcified aorta. In this technique, the saphenous veins are longer than normal, which may cause graft failures in the long term. Studies that investigate graft patencies in the mid and long term are necessary. Graft patencies in our study were similar to those in the literature.

## Conclusions

We believe that when a severely atherosclerotic ascending aorta is encountered during CABG procedures, techniques that do not require cross-clamping, partial clamping or manupilation of the aorta, such as the beating heart with or without supportive CPB and a fibrillating heart, should be preferred. In summary, we conclude that in patients with a severely calcified (porcelain) ascending aorta, in addition to the use of IMA grafts using the "no-touch" technique and placing proximal anastomoses on one of the branches of the aortic arch instead of the aorta in an extra-anatomic fashion are straightforward and useful strategies to reduce the perioperative risk of cerebrovascular events due to atheroembolism.

## Competing interests

The authors declare that they have no competing interests.

## Authors’ contributions

GS designed the study, performed data collection, analysis and interpretation and drafted the article. KS performed critical revisions and provided the final approval of the article. MK assisted in data interpretation and performed critical revisions of the article. ED assisted in data interpretation and performed critical revisions of the article. All authors read and approved the final manuscript.
